# Thermodynamics and Kinetics of Guest-Induced Switching between “Basket Handle” Porphyrin Isomers

**DOI:** 10.3390/molecules19045278

**Published:** 2014-04-23

**Authors:** Alexander B. C. Deutman, Tim Woltinge, Jan M. M. Smits, René De Gelder, Johannes A. A. W. Elemans, Roeland J. M. Nolte, Alan E. Rowan

**Affiliations:** Institute for Molecules and Materials, Radboud University Nijmegen, Heyendaalseweg 135, Nijmegen 6525 AJ, The Netherlands

**Keywords:** molecular switches, porphyrins, host-guest chemistry, viologens

## Abstract

The synthesis and switching properties of two “basket handle” porphyrin isomers is described. The *cis*-oriented *meso*-phenyl groups of these porphyrins are linked at their *ortho*-positons via benzocrown-ether-based spacers, which as a result of slow atropisomerization are located either on the same side of the porphyrin plane (*cis*), or on opposite sides (*trans*). In solution, the *cis*-linked isomer slowly isomerizes in the direction of the thermodynamically more stable *trans*-isomer. In the presence of viologen (*N,N'*-dialkyl-4,4'-bipyridinium) derivatives, which have different affinities for the two isomers, the isomerization equilibrium could be significantly influenced. In addition, the presence of these guests was found to enhance the rate of the switching process, which was suggested to be caused by favorable interactions between the positively charged guest and the crown ethers of the receptor, stabilizing the transition state energies of the isomerization reaction between the two isomers.

## 1. Introduction

Host-guest chemistry provides a valuable platform for studying binding processes observed in nature with the help of relatively simple mimics. Many artificial receptors have been designed, displaying similar features as observed in natural host-guest systems, such as induced-fit binding [1,2,3,4], lock and key mechanisms [5], and cooperative binding effects [6,7,8,9,10,11,12]. Dynamic recognition processes, in which the guest influences the conformation of the receptor, play a crucial role in biological systems and are of particular importance for allosteric binding, regulation or feedback [13,14,15,16,17]. To date, there are a number of examples of artificial dynamic recognition systems that exhibit complexation-induced atropisomerism or conformational changes [18,19,20,21,22,23,24,25,26,27,28,29,30]. The vast majority of these studies, however, is focused on thermodynamic aspects of complex formation, whereas kinetic aspects, while being of significant importance for determining the actual binding mechanisms involved, are not explored. Houk *et al.* have convincingly demonstrated that the origin of binding affinities between guests and receptors (ranging from small synthetic cavitands to large proteins) are well-understood, and that enzymes do not show any special binding behaviour for their substrates when compared to artificial receptors [31]. This is a powerful reminder that the ability to catalyze reactions arises from transition state interactions and not from substrate binding. Therefore, in order to get a better understanding of the combination of interactions responsible for the nature of transition states in enzymatic systems, it is of key importance to also study the transition states of relatively simple artificial host-guest systems. However, for many of these systems the kinetics of the binding processes are simply too fast to be studied accurately with the help of general methods.

In order to gain detailed insight in the kinetic aspects of complexation-induced conformational changes of dynamic artificial receptors, we have designed a porphyrin-based receptor molecule that interconverts extremely slowly between two distinct conformational isomers. Thanks to this slow exchange, not only the guest-induced kinetics of interconversion between the two isomers could be studied accurately, but in addition also the binding affinities of each of the two isomers towards viologen (*N,N'*-dialkyl-4,4'-bipyridinium) guests could be determined. A complete kinetic and thermodynamic picture of a guest-induced “switching” was obtained, which revealed that the presence of guests not only influences the thermodynamic outcome, as expected on the basis of the obtained individual equilibrium constants, but also significantly influences the kinetics of the switching process.

## 2. Results and Discussion

### 2.1. Design

**Scheme 1:**
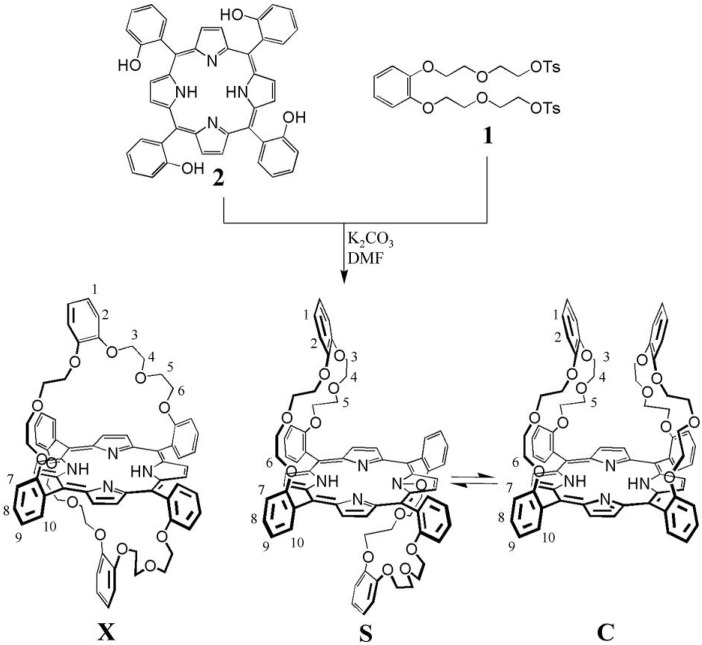
Synthesis of basket handle isomers **X**, **S** and **C**.

We decided to make use of the atropisomerization properties of *ortho-meso-*phenyl-substituted porphyrins in the design of the receptor. It is known that the restricted rotation of the aryl rings in many of such porphyrins can be extremely slow [32,33] (rates between 10^−4^ and 10^−9^ s^−1^ depending on the substitution pattern and metal ion present in the porphyrin), which often allows the isolation of the individual atropisomers. By appending two adjacent-linked binding pockets (“handles”) to the porphyrin *ortho*-phenyl positions, two so-called “basket handle” porphyrin [34,35,36,37,38,39,40] isomers (*trans*-linked **S** and *cis*-linked **C**; Scheme 1), which can interconvert slowly via atropizomerization, requiring the rotation around two aryl rings, were obtained By appending crown ether handles that are specifically designed for the complexation of viologen derivatives, the adjacent-linked “basket handle” porphyrins are expected to become excellent receptor molecules for these guests. The *cis*-linked isomer **C** was expected to have higher affinity for the viologen derivatives than *trans*-linked isomer **S** as a result of the interplay of two crown ether handles on the same side of the porphyrin plane in the former isomer. The addition of viologen derivatives should therefore result in the slow switching of the equilibrium in the direction of isomer **C**, a process that allows the accurate determination of both the thermodynamics and the kinetics involved.

### 2.2. Synthesis

**Figure 1:**
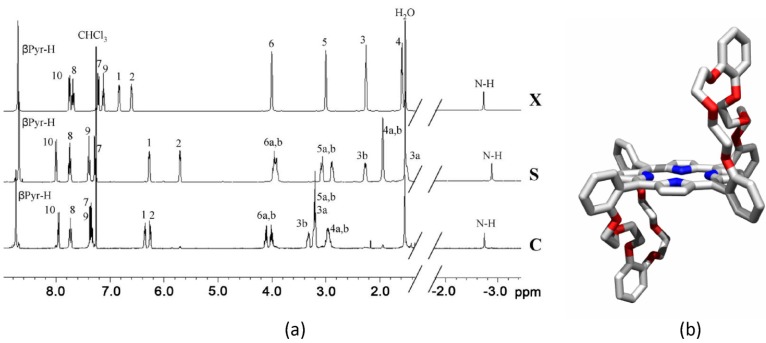
(**a**) ^1^H-NMR spectra (400 MHz) of “basket handle” porphyrin isomers **X**, **S** and **C** in CDCl_3_ with proton assignments based on COSY and 2D-ROESY NMR experiments. See Scheme 1 for proton numbering. (**b**) X-ray structure of isomer **S** (protons have been omitted for clarity).

A mixture of “basket handle” porphyrin isomers **X**, **C** and **S** (Scheme 1) was prepared in an overall yield of 52% by reacting ditosylate **1** [41] with 5,10,15,20-tetrakis(*meso*-*o*-hydroxyphenyl)porphyrin **2** [42] under basic conditions in DMF at 110 °C. The three different isomers could be separated by preparative TLC and were obtained in a ratio **X**:**S**:**C** = 4:8:1. The individual isomers were identified with the help of ^1^H-NMR spectroscopy in CDCl_3_ (Figure 1a), and in addition the X-Ray structure of the **S**-isomer was determined (Figure 1b). Isomer **X** has an S_2_ symmetry, and as a result the ^1^H-NMR spectrum revealed only four resonances for all the 32 crown ether protons H-3, H-4, H-5 and H-6, and one single resonance for the *β*-pyrrolic porphyrin protons. Isomers **S** and **C** have C_2h_ and C_2v_ symmetry, respectively, and as a result the crown ether proton resonances show AB-patterns in the ^1^H-NMR spectra and two distinct resonances for the *β*-pyrrolic protons. The resonances of the protons of the handles of **S** (H-1–H-6) are significantly shifted upfield in comparison to those of **C**, which indicates that they experience more shielding from the porphyrin ring-current. This can be understood since in **S** there is space to position both handles in the proximity of the porphyrin, whereas in **C** this is sterically impossible. Also the pyrrole NH resonances of **S** are shifted upfield compared to those of **C**, which suggests that the ring currents of the phenyl groups of the handles shield the center of the porphyrin more in **S** than in **C**. These combined observations indicate that, unlike in the X-ray structure of **S** in which the handles bend away from the porphyrin plane (Figure 1b), in solution they are on average folded over the porphyrin plane. This folding was confirmed by a 2D-ROESY NMR measurement which showed nOe contacts between the handle phenyl protons H-1 and H-2 and the β-pyrrole protons and H-10, respectively.

### 2.3. Isomerization

**Figure 2:**
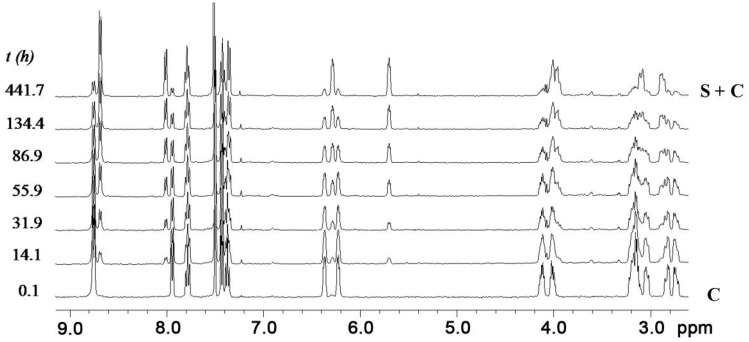
Series of partial 400 MHz ^1^H-NMR spectra in time revealing the decrease in abundance of *cis*-linked isomer **C** in favor of the thermodynamically more stable *trans*-linked isomer **S** at 25 °C in CDCl_3_/CD_3_CN 1:1 (*v*/*v*).

As observed in other adjacent-linked “basket handle” porphyrins [36] the adjacent-*cis*-linked isomer **C** and the adjacent-*trans*-linked isomer **S** slowly interconvert in time. Figure 2 reveals that upon standing, the resonances of **S** in the ^1^H-NMR spectra increase in intensity at the expense of those of **C**. Isomer **S** is thermodynamically significantly more stable than isomer **C**, since equilibrium is reached at a ratio of **S**/**C** = 7.6. This observation is in contrast with results in a previous report, in which the switching of a hexyl-bridged basket-handle porphyrin resulted in equimolar amounts of the adjacent-cis-linked and adjacent-trans-linked isomers at equilibrium [43], as would statistically be expected. The rate constants for the isomerization process (*k*_C→S_) could be simply determined by first order analysis of the decrease in the relative intensities [44] of the resonances of **C** in time.

The equilibrium constants (*K*_S/C_ = [**S**]_eq_/[**C**]_eq_) were obtained at different temperatures from the ratio between **S** and **C** at equilibrium, after which the rate constant *k*_S→C_ could be calculated indirectly from *K*_S/C_ = *k*_C→S_/*k*_S→C_. Isomerization experiments in which the switching from **S** to **C** was monitored provided identical values for *k*_S→C_ as were obtained by the indirect method via *K*_S/C_ and *k*_C→S_. The calculated constants are presented in Table 1.

**Table 1:** Calculated rate constants for the switching of **C** to **S** (*k*_C→S_) and for the switching of **S** to **C** (*k*_S→C_) at different temperatures, and the equilibrium constant *K* that represents the ratio of **S**/**C** at equilibrium.

*T* (°C)	*k*_C→S_ (s^−1^) *^a^*	*k*_S→C_ (s^−1^) *^b^*^,*c*^	*K* *^b^*
25	2.8 × 10^−6^	3.7 × 10^−7^	7.6
32.5	6.0 × 10^−6^	1.0 × 10^−6^	5.8
40	1.5 × 10^−5^	2.8 × 10^−6^	5.3
47.5	3.2 × 10^−5^	6.8 × 10^−6^	4.7

*^a^* Estimated error 5%; *^b^* Estimated error 15%; *^c^* Calculated from *k*_S→C_ = *k*_C→S_/*K*.

**Figure 3:**
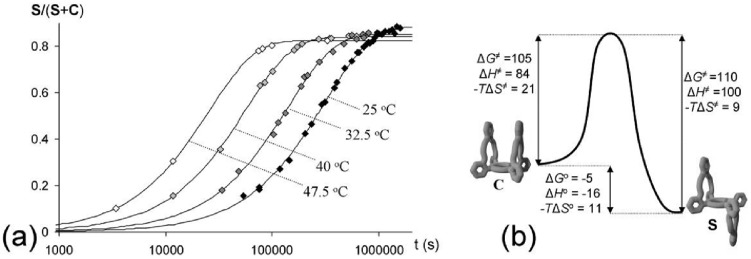
(**a**) Kinetics of switching from **C** to **S** at different temperatures in CDCl_3_/CD_3_CN 1:1 (*v*/*v*), monitored by ^1^H-NMR. (**b**) Energy diagram of the switching process with the entropic and enthalpic parameters (all in kJ/mol).

The switching from **C** to **S** was monitored at four different temperatures (Figure 3a) from which, with the help of van’t Hoff and Eyring plots, the entropic (Δ*S*) and enthalpic (Δ*H*) contributions to the activation energy (Δ*G*^≠^) and the free energy of binding (Δ*G*°) could be determined. The resulting energy diagram with all these parameters is presented in Figure 3b. The switching process is unfavorable both in entropy and in enthalpy, but the majority of the free energy of activation (Δ*G*^≠^) is enthalpic in origin (the proposed mechanism will be presented later).

The obtained parameters Δ*S*° and Δ*H*° (Figure 3b) revealed that the formation of the *trans*-linked isomer **S** is enthalpically more favorable than the *cis*-linked isomer **C**, whereas the latter is entropically more likely to form. The value of Δ*H*° suggests that **S** experiences more stabilizing intramolecular interactions than **C**. These are presumably π-π interactions between the phenyl groups of the handles and the porphyrin plane, and additional van der Waals interactions. The entropic parameters suggest that **C** is more disordered than **S**, which could be the result of a restricted motion of the handles in **S** as a result of the intramolecular binding interactions (entropy-enthalpy compensation [45]). Another possibility is that **S** needs to organize more solvent molecules in a defined shell than **C**, which would result in the release of solvent to the bulk upon switching from **S** to **C**, thereby accounting for the entropic gain. The observation that **C** dissolves significantly better in the used solvent mixture than **S** could validate this hypothesis, although solubility depends on more factors.

### 2.4. Binding of Viologen Derivatives

**Figure 4:**
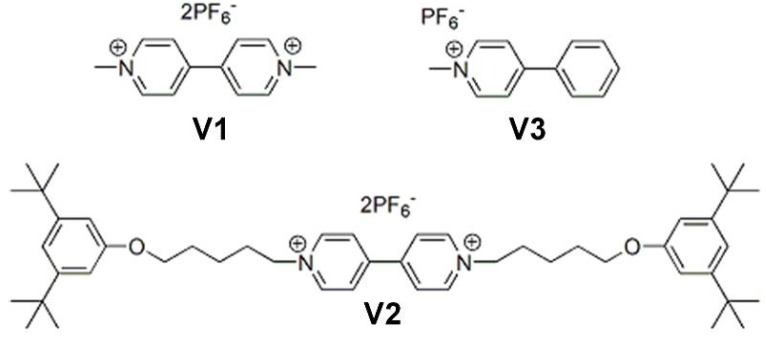
Structure of viologens **V1** and **V2** and pyridinium compound **V3**.

The binding of viologen derivatives **V1**, **V2**, and **V3** (Figure 4) to isomers **C** and **S** was investigated. As a result of the high kinetic stabilities of **C** and **S**, no detectable isomerization was observed in the first few hours after their separation, which allowed the study of the binding properties of the individual isomers. A ^1^H-NMR titration between **C** and **V1** revealed that **V1** binds strongly to this receptor in a face-to-face geometry with respect to the porphyrin [46]. The addition of increasing amounts of **V1** to **C** resulted in large downfield complexation induced shift (CIS) values of the crown ether proton resonances (H-3 to H-6), which indicates that their position in the proximity of the porphyrin is replaced by **V1** (Table 2). The pyrrole NH resonances of **C** shifted dramatically upfield upon complex formation, indicating their shielding by the aromatic rings of **V1**. Also the signals of **V1** displayed large upfield shifts compared to their original positions, confirming their position in the proximity of the shielding porphyrin ring current. The addition of one equivalent of **V1** resulted in the full binding to **C**, stressing the strong affinity between the components. To derive an accurate value for the association constant (*K*_CV1_), the binding of **V1** to **C** was investigated at lower concentrations (**[C]** ≈ 10^−6^ M) with the help of a fluorescence titration. The addition of increasing amounts of **V1** to a solution of **C** resulted in the quenching of the porphyrin fluorescence of the receptor. The obtained binding curve could be fitted with a 1:1 binding isotherm and the association constant was calculated to be *K*_CV1_ = 3 × 10^5^ M^−1^ (Table 3).

**Table 2:** Selected calculated CIS values (ppm) of receptor proton signals upon binding of viologens to **S** and **C**. *^a^*

Proton *^b^*	C			S		
	V1	V2	V3 *^d^*	V1	V2 *^e^*	V3 *^d^*
H-1	0.00	0.01	0.01	−0.28	−0.06	−0.28
H-2	−0.03	−0.10	−0.03	−0.13	−0.08	−0.06
H-3 to H-5 *^c^*	0.42	0.43	*^f^*	0.47	*^f^*	0.50
H-6a	0.51	0.64	*^f^*	0.25	*^f^*	0.21
H-6b	0.47	0.29	*^f^*	0.21	*^f^*	0.17
N-H	−1.04	−0.98	−0.51	−1.12	−0.33	−0.85

*^a^* Calculated from ^1^H-NMR experiments (400 MHz, 298 K, CDCl_3_/CD_3_CN 1:1 (*v*/*v*)); *^b^* See Scheme 1 for proton numbering; *^c^* Average shifts of the series of protons; *^d^* Concentration **V3**: 1 × 10^−2^ M; *^e^* Concentration **V2**: 3.3 × 10^−3^ M; *^f^* Resonances were obscured.

**Table 3:** Association constants (*K*_a_) and binding free energies (Δ*G*°) at 298 K and the enthalpic (Δ*H*°) and entropic (Δ*S*°) contribution to the binding free energy between isomers **C** and **S** and viologen guests.

Receptor isomer	Guest	*K*_a_*^c^*(M^−1^)	Δ *G*°(kJ/mol)	Δ *H*° *^d^*(kJ/mol)	Δ *S*° *^e^*J/molK
**S**	**V1** ( *K*_SV_)	1.3 × 10^4^ *^a^*^,*b*^	−23.6	−31.3	−25.9
	**V1** ( *K*_VSV_)	6.0 × 10^2^ *^a^*	−15.8		
**C**	**V1**	3.0 × 10^5^ *^b^*	−31.2	−30.1	+3.7
	**V2**	3.5 × 10^3^ *^a^*	−20.2		

*^a^* Determined in CDCl_3_/CD_3_CN 1:1 (*v*/*v*) by ^1^H-NMR titrations; *^b^* Determined In CHCl_3_/CH_3_CN 1: (*v*/*v*) by fluorescence titrations; *^c^* Estimated error 30%; *^d^* Estimated error 10 kJ/mol; *^e^* Estimated error 10 J/molK.

**Figure 5:**
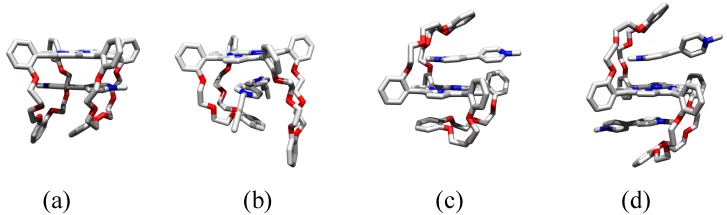
Molecular models, based on the binding studies, of the 1:1 complexes between (**a**) **C** and **V1**, (**b**) **C** and **V2** (blocking groups are not shown for clarity), (**c**) **S** and **V1** and (**d**) the 1:2 complex between **S** and **V1**.

Molecular modeling revealed that **V1** preferably accommodates its positive charges in between the two “handle” crown ether rings of **C**. The complex adopts a so called suit[2]ane [47] geometry in which the positive charges are wrapped inside the crown ether sleeves of the suit-shaped receptor **C** (see Figure 5a). Since it is impossible to directly derive the exact geometry of the complex with the help of NMR techniques, and attempts to obtain an X-ray structure of the complex were unsuccessful, it was decided to compare the binding between **C** and **V1** with that between **C** and **V2**. **V2** cannot adopt the proposed suit[2]ane geometry as a result of the presence of 3,5-di-*tert*-butylphenyl blocking groups, which can impossibly slip through the crown ether rings of **C**. A ^1^H-NMR titration between **C** and **V2** indicated that **V2** adopts a different binding geometry with **C** than **V1**. Although **V2** also binds in a face-to-face orientation with respect to porphyrin plane of **C**, as can be concluded from the observed upfield shift of the pyrrole NH proton resonances of the receptor (Table 2), the CIS values for all the other proton resonances are significantly different for the complex between **C** and **V2** when compared to the complex between **C** and **V1**. Moreover, the association constant for the binding of **V2** to **C** is almost two orders of magnitude lower than that for the binding of **V1** to **C** (Table 3). These combined results strongly suggest that **V1** indeed binds to **C** in the proposed suit[2]ane geometry, whereas **V2** adopts a geometry as indicated in Figure 5b.

In addition to the binding to **C**, the binding of **V1** to *trans*-linked **S** was investigated. A ^1^H-NMR titration revealed complex formation between **V1** and **S**, which was expressed in large CIS values for the proton resonances of **V1** and **S**. As observed for the binding of **V1** with **C**, the pyrrole NH proton resonances and aromatic viologen signals shifted upfield, whereas the crown ether proton resonances shifted downfield (Table 2), which suggests a binding geometry in which the porphyrin ring of **S** and the aromatic rings **V1** adopt a face-to-face orientation (Figure 5c). The experimentally obtained binding curve could however not be fitted with a 1:1 binding isotherm, which strongly points in the direction of 1:2 complex formation between **S** and **V1**. This was moreover indicated by the CIS value of the pyrrole NH proton signals of **S** (Table 2). These signals shifted significantly further upfield upon complexation of **V1** than in the complex between **V1** and **C** (and other known face-to-face viologen-porphyrin complexes known in literature [41,48,49]), which indicates that this is the result of the presence of two viologen molecules that are sandwiching the porphyrin (Figure 5d). Data analysis of the binding curves with a 1:2 host-guest binding isotherm gave a satisfying fit with association constants of 1.3 × 10^4^ M^−1^ and 6 × 10^2^ M^−1^ for the binding of the first (*K*_SV_) and second (*K*_VSV_) molecule of **V1** to **S**, respectively. Considering the statistical factor of 4 for the difference in binding of the first and the second identical guest molecule to a bivalent receptor, there is a negative cooperative effect for the binding of the second molecule of **V1** to **S** with an α-value of 0.18. This is most probably caused by the repulsive interactions between the positive charges of the two viologen derivatives [50].

Because the guest-induced switching between the conformers, which will be presented in the following sections, is performed at elevated temperatures, the effect of temperature on the binding constants of **V1** to **S** and **C**, respectively, was studied with the help of fluorescence titrations. Although **V1** was shown to form 1:2 host-guest complexes with **S** at ^1^H-NMR concentrations (10^−3^ M), at the used experimental concentrations of the fluorescence titrations (10^−6^ M) 1:2 complex formation was so marginal that it could be ignored, and the obtained fluorescence titration curves could therefore be fitted with the use of simple 1:1 binding isotherms. This was emphasized by both the good fits and the calculated value of the association constant that were obtained from the fluorescence titration for the 1:1 complex between **V1** and **S**, which was in very good agreement with the value of *K*_SV_ obtained from the ^1^H-NMR titration experiment at the same temperature [51]. With the use of Van het Hoff plots, the enthalpic (Δ*H*°) and entropic (Δ*S*°) contributions to the total free binding energy (Δ*G*°) of **V1** with **S** and **C**, respectively, could be determined. Quite surprisingly, the calculated parameters revealed that the differences in binding strength between **V1** and **C** and **V1** and **S**, respectively, are mainly entropic in origin (Table 3). Similar values were obtained for the binding enthalpy (Δ*H*°). Complex formation between **C** and **V1** is slightly favorable in entropy, whereas complex formation between **S** and **V1** is unfavorable in entropy. 

Intuitively, two good reasons can be envisaged why the binding of **V1** to **C** should be enthalpically more favorable than the binding of **V1** to **S**. The first reason is that isomer **C** can provide more stabilizing interactions to **V1** than isomer **S** as a result of the presence of two crown ether handles, which can both interact with the viologen guest, on the same side of the porphyrin plane. The second reason is that within isomer **S** more intramolecular interactions are present than within **C**, which have to be overcome in order to accommodate **V1** (as suggested by the value of Δ*H*° for the switching process as presented above). The fact that no enthalpic difference is observed between the two binding processes, and that the difference in binding is mainly entropic in origin, therefore suggests that the binding of viologen derivatives to both isomers depends to a crucial extent on the desolvation of the viologen derivatives upon complex formation. Isomer **C** can fully accommodate the positive charges of the viologen, thereby perfectly shielding it from the solvent (Figure 5a). In contrast, isomer **S** is only capable of shielding one of the positive charges of the viologen from the solvent (Figure 5c). As a result, the binding of **V1** to **C** is accompanied by the release of more solvent molecules to the bulk than the binding of **V1** to **S**, which accounts for the observed difference in binding entropy. 

### 2.5. Guest-Induced Switching: Thermodynamics

As a result of the higher affinity of **V1** for **C** than for **S** (∆∆*G*° = 7.6 kJ/mol), it would be expected that in the presence of the guest the equilibrium should be switched further in the direction of the thermodynamically less favorable isomer **C**. Because **S** is 5 kJ/mol more stable than **C** (Figure 4b), it could theoretically result in an equilibrium situation which has its free energy shifted maximally 2.6 kJ/mol in the direction of **C**, and which would be translated into a ratio of approximately 74% **C** and 26% **S** at equilibrium, or [**S**]_tot-eq_/[**C**]_tot-eq_ = 0.36. A first isomerization experiment, in which a mixture of **S** and **V1** was annealed at 80 °C in toluene/acetonitrile 1:1 (*v*/*v*) for 12 h, indeed revealed that the conformational equilibrium had shifted in the direction of **C**. After workup and purification of the reaction mixture, the different isomers were isolated in a ratio of approximately **C**:**S** = 2:1. This ratio is strikingly different from the ratio obtained after annealing isomer **S** under the same experimental conditions but in the absence of **V1**, which amounted to **C**:**S** ≈ 1:5.

**Figure 6:**
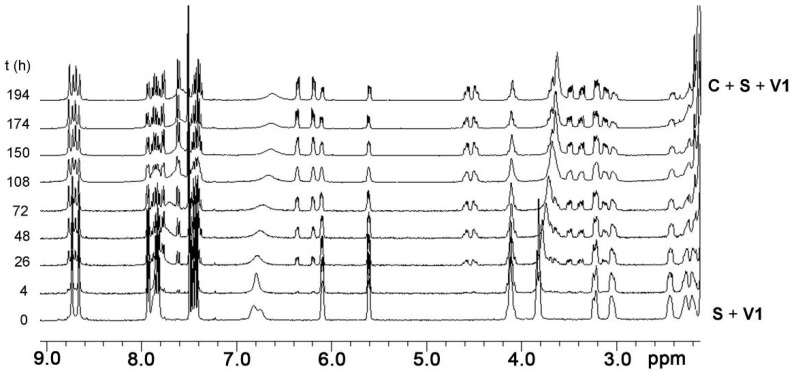
Series of partial 400 MHz ^1^H-NMR spectra recorded over time, revealing the decrease in population of isomer **S** at the expense of the population of isomer **C** in the presence of 1.3 equiv. of **V1** at 37.5 °C in CDCl_3_/CD_3_CN 1:1 (*v*/*v*).

In order to gain more detailed information about the guest-induced switching, it was decided to follow the switching from **S** to **C** in the presence of **V1** with the help of ^1^H-NMR spectroscopy. The ^1^H-NMR spectra of a mixture of **S** (1 mM) and **V1** (1.3 mM) in CDCl_3_/CD_3_CN 1:1 (*v*/*v*) at 37.5 °C revealed that in time isomer **C** was formed at the expense of isomer **S** (Figure 6), resulting in a final equilibrium ratio of [**S**]_tot-eq_/[**C**]_tot-eq_ = 0.56 (64% **C** and 36% **S**). This ratio is different from the ratio (0.36) mentioned above because of the different solvent mixture and the different temperature.

**Figure 7:**
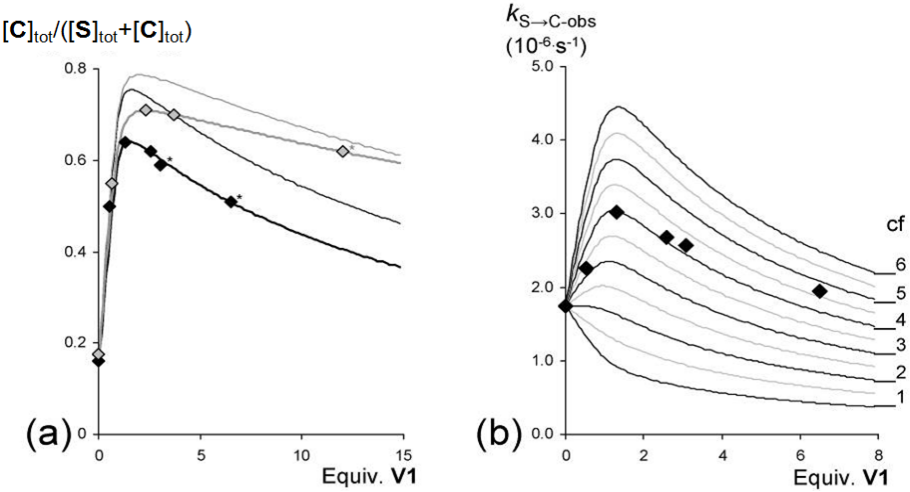
(**a**) Equilibrium ratios ([**C**]_tot_/([**S**]_tot_ + [**C**]_tot_}) at 37.5 °C (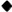) and 47.5 °C (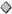) plotted against the concentration of **V1** present in solution and the expected equilibrium ratios ([**C**]_tot_/([**S**]_tot_ + [**C**]_tot_}) based on the experimentally derived individual equilibrium constants (thin lines) and the bests fits (thicker lines matching the data points) of the experimental data (37.5 °C: *K*_S/C_ = 5.5, *K*_SV_ = 1.5 × 10^4^ M^−1^, *K*_VSV_ = 2.0 × 10^2^ M^−1^, *K*_CV_ = 1.8 × 10^5^ M^−1^. 47.5 °C: *K*_S/C_ = 4.7, *K*_SV_ = 7.0 × 10^3^ M^−1^, *K*_VSV_ = 70 M^−1^, *K*_CV_ = 9.5 × 10^4^ M^−1^). (**b**) Observed initial rates (*k*_S→C-obs_) for the switching of **S** to **C** in solutions with different concentrations of **V1** at 37.5 °C. The framework represents the theoretical rates based on different values of the cooperativity factor (*cf*), assuming equilibrium constants *K*_SV_ = 1.5 × 10^4^ M^−1^ and *K*_VSV_ = 2.0 × 10^2^ M^−1^.

**Figure 8:**
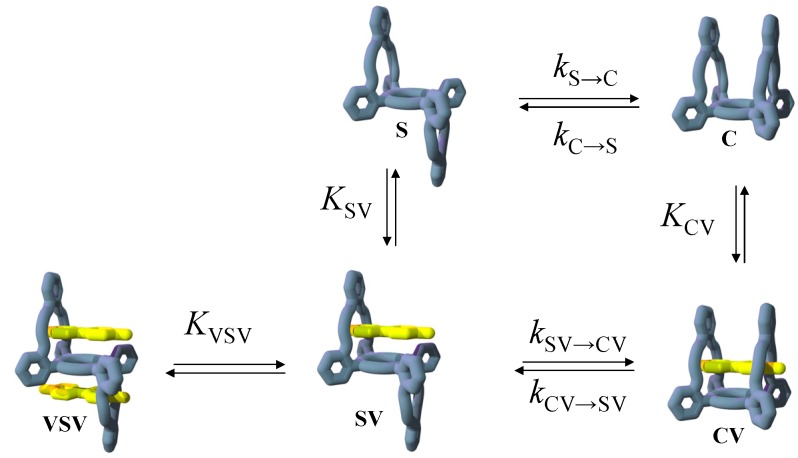
Binding scheme of the isomerization between **S** and **C** in the presence of **V1**.

To further explore the switching effect of **V1**, the isomerization from **S** to **C** in solutions containing varying concentrations of this guest was monitored by ^1^H-NMR spectroscopy. The measurements revealed that there is an optimal concentration of **V1** in which the equilibrium ratio ([**C**]_tot_/{[**C**]_tot_ + [**S**]_tot_}) is shifted furthest in the direction of [**C**]_tot_ (Figure 7a and Table 4), after which increasing concentrations of **V1** resulted slowly in the shifting back of the conformational ratio in the direction of [**S**]_tot_. This observation is attributed to the possibility of also forming the 1:2 complex between **S** and **V1**, as presented in Figure 8. At relatively low concentrations of **V1**, the equilibrium will shift towards the 1:1 complex between **V1** and **C** (since *K*_CV_ > *K*_SV_) and thus in the direction of [**C**]_tot_. At further increasing concentrations of **V1**, however, the formation of the 1:2 complex between **S** and **V1** will become more favorable, which results in a shifting back of the equilibrium situation towards [**S**]_tot_. This behavior follows directly from Equation (1), which presents the equilibrium constant (*K*_switch_) as a function of free **V1** in solution ([**V**]) and all the individual equilibrium constants:

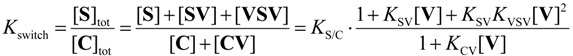
(1)

In Equation (1), *K*_S/C_ is the equilibrium constant between **S** and **C** in the absence of viologen guests, *K*_SV_ and *K*_CV_ are the association constants of **V** to **S** and **C**, respectively, for the formation of the 1:1 complexes, and *K*_VSV_ is the association constant for the formation of the 1:2 complex between **S** and **V**. The concentration of free **V** is obviously directly related to the concentration of isomers **C** and **S** in solution, but it is clear from Equation (1) that there will exist a concentration of **V** in which *K*_switch_ has a minimum value and thus that [**C**]_tot_ will be maximal. The value for *K*_switch_ as a function of different equilibrium constants and concentrations of **V1** could be determined numerically with the use of Mathematica^®^. Although the experimental data did not exactly match the theoretical calculations on the basis of all the experimentally derived individual equilibrium constants, as can be seen in Figure 7a (which is not surprising, since four equilibrium constants are involved, some of which have errors of up to 30% and *K*_VSV_ had to be estimated), the trend in switching is clearly as would be expected on the basis of the binding model in Figure 8. Moreover, the data could be fitted with the assumed binding model and the calculated equilibrium constants as obtained by the fit did not deviate significantly from the experimentally derived values for the individual equilibrium constant (see Figure 7a).

**Table 4:** Calculated rate constants (*k*_S→C-obs_) and equilibrium constants (*K*_switch_) for the switching of **S** to **C** in solutions with different concentrations of viologen guests **V1**−**V3**. *^a^*

Guest	Conc (mM)	*k*_S→C-obs_*^b,d^* (s^−1^)	*K*_switch_ *^c,e^*
**No**	0	1.8 × 10^−6^	5.5
**V1**	0.5	2.3 × 10^−6^	1.0
**V1**	1.3	3.0 × 10^−6^	0.6
**V1**	2.6	2.7 × 10^−6^	0.6
**V1**	3.1	2.6 × 10^−6^	0.7
**V1**	6.5	1.9 × 10^−6^	1.0
**V2**	3.3	2.4 × 10^−6^	0.9
**V3**	20	1.1 × 10^−6^	10.0

*^a^* Determined by ^1^H-NMR experiments in CDCl_3_/CD_3_CN 1:1 (*v*/*v*) at 37.5 °C; *^b^* Calculated from the initial 25% of the switching curves; *^c^* Calculated from the ratio [**S**]_tot_/[**C**]_tot_ at equilibrium; *^d^* Estimated error 20%; *^e^* Estimated error 15%.

**Figure 9:**
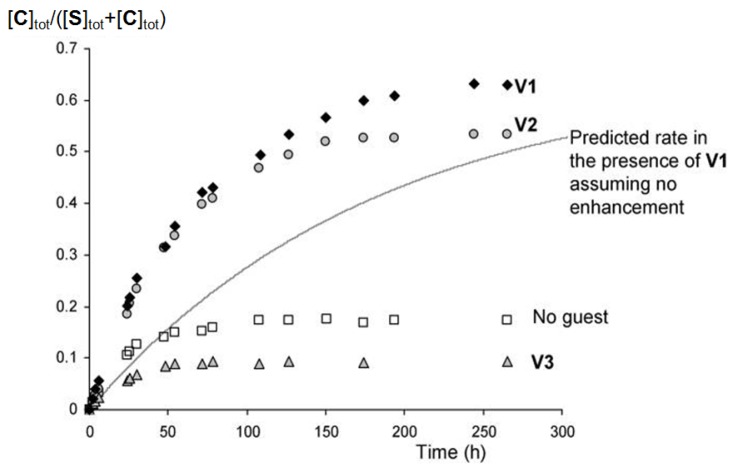
Kinetics of the switching of **S** (1 × 10^−3^ M) to **C** in the absence of guest (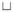) and in the presence of 1.3 equivalents of **V1** (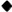), 3.3 equivalents of **V2** (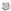) and 20 equivalents of **V3** (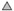). The line represents the calculated curve for the switching in the presence of 1.3 equivalents of **V1** assuming no induced switching effect.

In addition to experiments in which switching was induced by **V1**, also experiments were carried out in which switching was triggered by the doubly blocked guest **V2** and the guest **V3** (1-methyl-4-phenylpyridinium hexafluorophosphate). As can be seen in Figure 9, also **V2** is capable of switching the equilibrium in the direction of **C**, which indicates that this guest has a higher affinity for **C** than for **S**. This binding process was further confirmed by the gradually upfield shifting aromatic resonances of the guest in time upon switching from **S** to **C** in the ^1^H-NMR spectra. Although the binding of **V2** to **S** was not studied in detail, it is obvious that **C** can provide more stabilizing interactions (or cause more solvent shielding upon complex formation) to **V2** than **S**. The switching experiment in the presence of **V3** (20 equiv.) revealed that this guest is not capable of switching the equilibrium in the direction of [**C**]_tot_. Instead, it causes the opposite effect and the equilibrium is shifted more in the direction of [**S**]_tot_ (Figure 9). This observation suggests that **S** has a higher affinity for **V3** than **C**, which is more or less in line with expectation since only one handle is needed for the accommodation of the single positive charge of **V3** [52].

### 2.6. Guest-Induced Switching: Kinetics

In addition to the thermodynamics we also investigated the kinetics of the guest-induced switching. The observed initial rates (*k*_S→C-obs_) in solutions with different concentrations of **V1** at 37 °C were calculated from the initial 25% of the switching curves, as obtained by ^1^H-NMR spectroscopy. The data, which are presented in Figure 7b and Table 4, reveal that the observed initial rates increase upon increasing concentrations of **V1**, and reach a maximum value after which they decrease again. This decrease is fully in line with expectation, since increased concentrations of **V1** shift the initial equilibrium further in the direction of the 1:2 host-guest complex **VSV** (Figure 8) in which both handles are restricted from rotation. The initial increase in rate in solutions with lower concentrations of **V1**, on the other hand, is surprising and suggests that the switching is enhanced by the presence of **V1**. Statistically, it can be expected that the switching rate from **S** to **C** in the presence of **V1** (*k*_SV→CV_, see Figure 9) is a factor 2 lower than in the absence of **V1** (*k*_S→C_) as a result of the restricted rotation of one of the handles of **S** upon complex formation with **V1**. If a viologen-induced rate enhancement is not taken into account, the observed switching rates (*k*_S→C-obs_) should therefore decrease upon complex formation between **V1** and **S**, and drop even further upon a further increase in concentration of **V1**, as a result of 1:2 host-guest complex formation. In order to be able to fit the experimental data, a rate enhancement factor (*cf*) has to be included into the model. The initial rate (*k*_S→C-obs_) should evolve as a function of [**S**], [**SV**] and [**VSV**] according to Equation (2), in which *k*_SV→CV_ is defined according to Equation (3) in which both the statistical factor of 0.5 and the rate enhancement factor is taken into account: 

*k*_S→C-obs_ = *k*_S→C_·[S]/[S]_tot_ + *k*_SV→CV_·[SV]/[S]_tot_ (2)

*k*_SV→CV_ = *cf*·0.5·*k*_S→C_ (3)

*k*_SV→CV_ = *cf*·0.5·*k*_S→C_·*K*_SV_/*K*_CV_ (4)

Since the individual constants *k*_S→C_, *K*_SV_ and *K*_VSV_ were determined separately (see above), the magnitude of the factor *cf* could be calculated. To this end, the expected overall initial rate constants were calculated with the help of Mathematica^®^ as a function of the magnitude of *cf* and the concentration of **V1** (which determines the ratios of [**S**], [**SV**] and [**VSV**]). The calculated rates as a function of **V1** are presented as the framework in Figure 7b, and from this framework it can be concluded that the magnitude of *cf* is approximately 4, hence the switching rate is enhanced in the presence of **V1** by a factor of 4. In order to stress the acceleration of the switching process in the presence of **V1**, the expected kinetic curve of switching from **S** to **C** under the same conditions, assuming *cf* = 1, is presented in Figure 9. In addition to **V1**, also **V2** clearly accelerates the switching process, as can be observed in Figure 9 and Table 4. The observed rate (*k*_S→C-obs_) in the presence of **V3**, on the other hand, is lower than *k*_S→C_ (Table 4), which indicates that the switching rate is not enhanced by **V3**.

### 2.7. Mechanism

**Figure 10:**
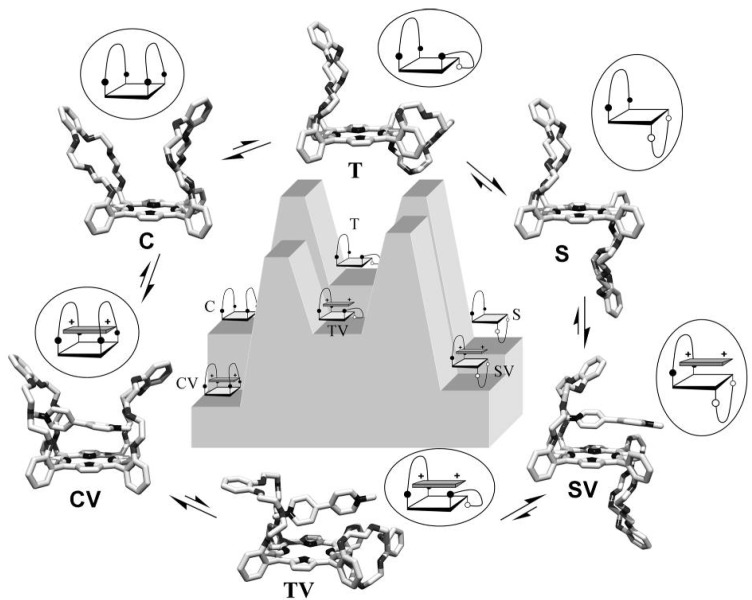
Proposed energy landscape of the switching between **C** and **S** in absence and in the presence of **V1**.

The magnitudes of the rate constants and the enthalpic and entropic contributions to the transition state energy for the switching process between the isomers **C** and **S** are all very similar to the values observed for atropisomerization of *ortho*-*meso*-phenyl-substituted porphyrins, involving rotations around one single bond [53,54,55,56,57,58,59]. This suggests that the mechanism of isomerization between **C** and **S** is not significantly different from other porphyrin atropisomerization processes. For this reason, the switching between **S** and **C** most probably does not occur not via simultaneous but via consecutive rotations around the two porphyrin *meso*-phenyl rings. As a result, the switching consists of two steps that have similar activation barriers, with in between them a local energy minimum in which the handle is halfway the switching process (**T** in Figure 10). The energy level of **T** is unfavorable compared to both the energy levels of **S** and **C** making that it cannot be observed experimentally. The presence of **V1** in the switching process is expected to have a significant influence on the relative energy levels. The same interactions that cause that the complex **CV** is energetically more favorable than the complex **SV** (Figure 10) will influence the transition state of the switching process. Transition state intermediate complex **TV** will experience additional stabilizing interactions compared to **T** as a result of the fact that half the handle is switched to the side of the viologen (hence [**T**]/[**S**] > [**TV**]/[**SV**]). Also the rotation around the second porphyrin *meso*-phenyl ring in the switching process from **T** to **C** is energetically more favorable as a result of these interactions. The viologen guest thus lowers the barriers associated with the switching process and effectively pulls the handle through to the other side of the porphyrin. Note that these same interactions should also cause a similar rate enhancement while switching back from **C** to **S**, in line with the principle of microscopic reversibility. The observation that both **V1** and **V2** (which binds in 90 degrees rotated geometries with respect to the porphyrin) accelerate the switching process is in line with this mechanism, since independent of the geometry of the eventual complexes, both **V1** and **V2** can exert these stabilizing interactions in the transition state. **V3** most probably accommodates its positive charge inside one crown ether handle and is consequently not capable of stabilizing the transition state involving the switching of the other handle, which accounts for the apparent absence of rate enhancement in the presence of **V3**. 

## 3. Experimental

### 3.1. Materials and Methods

All solvents and chemicals were used as received. K_2_CO_3_ was dried in an oven (150 °C). Chloroform and acetonitrile used in fluorescence titration experiments were distilled from CaCl_2_. Preparative TLC was performed on Merck (Darmstadt, Germany) silica glass plates (TLC Silica gel 60 F_254_). Fluorescence experiments were performed on a Perkin-Elmer LS50B luminescent spectrometer (Waltham, MA, USA) equipped with a thermostatted cuvette holder. UV-Vis spectra were recorded on a Cary 100 Conc UV-Vis spectrometer (Varian, Middelburg, The Netherlands). Maldi-TOF mass spectrometry was performed on a Bruker Biflex III spectrometer (Billerica, MA, USA). NMR spectra were taken on a Varian (Palo Alto, CA, USA) Inova 400 (400 MHz, ^1^H and 2D spectra) or on a Bruker (Billerica, MA, USA) DMX300 (75 MHz, ^13^C spectra) and calibrated to an internal standard of tetramethylsilane.The synthesis of ditosylate **1** was reported elsewhere [41]. The syntheses of 5,10,15,20-tetrakis(*meso*-*o*-hydroxyphenyl)porphyrin **2** [42], **V1** [41] and **V2** [60] are also described in the literature.

### 3.2. Syntheses

#### 3.2.1. “Basket Handle” Isomers X, S and C

A suspension of **1** (0.90 g, 1.51 mmol), 5,10,15,20-tetrakis(*meso*-*p*-hydroxyphenyl)-porphyrin **2** (0.50 g, 0.74 mmol) and K_2_CO_3_ (1 g, 7.2 mmol) in DMF (250 mL) was reacted for 16 h under an argon atmosphere at 110 °C. After filtration of the salts and evaporation of the solvents the product was purified by column chromatography (3% MeOH in CHCl_3_ (*v*/*v*)) yielding 450 mg (52%) of a mixture of isomers **X**, **S** and **C**. The three isomers could be separated by preparative TLC (5:5:1 toluene/ethyl acetate/acetonitrile (*v*/*v*/*v*)) to give the products in a ratio of **X**:**S**:**C** = 4:8:1. (adjacent-*trans*-linked **S** could also be selectively crystallized out of a mixture of 5:1 acetonitrile/chloroform (*v*/*v*) containing isomers **C** and **S**) The separate products were dissolved in a minimal amount of CHCl_3_ and to this solution *n*-pentane was added. This resulted in precipitates, which were collected by centrifugation and dried under vacuum at low temperatures yielding the different isomers **X**, **S** and **C** as purple solids. In order to prevent isomerization between **C** and **S**, both speed and low temperatures are of essential importance in the separation process.

Data for cross-*trans*-linked “basket handle” porphyrin isomer **X**: ^1^H-NMR (CDCl_3_ 400 MHz) δ 8.71 (s, 8H), 7.75 (d, 4H, *J* = 7.2 Hz), 7.69 (t, 4H, *J* = 7.9 Hz), 7.22 (d, 4H, *J* = 8.3 Hz), 7.12 (t, 4H, *J* = 7.4 Hz), 6.83 (m, 4H), 6.60 (m, 4H), 4.00 (M, 8H), 3.00 (M, 8H), 2.25 (t, 8H, *J* = 4.5 Hz), 1.59 (t, 8H, *J* = 4.6 Hz), −2.72 (s, 2H) ppm. ^13^C-NMR (CDCl_3_ 75 MHz) δ 158.68, 149.16, 135.02, 130.91, 129.69, 122.30, 119.60, 117.65, 115.65, 111.04, 69.65, 69.21, 68.63, 68.42 ppm. MALDI TOF *m*/*z* = 1180 (M + H^+^). UV-Vis (CHCl_3_) *λ*/nm (log(ε·M^−1^cm^−1^)) 418.5 (5.6), 513.0 (4.3), 546.5 (3.8), 589.0 (3.8), 643.5 (3.5).

Data for adjacent-*trans*-linked “basket handle” porphyrin isomer **S**: ^1^H-NMR (CDCl_3_ 400 MHz) δ 8.69 (s, 4H), 8.68 (s, 4H), 8.00 (dd, 4H, *J* = 1.7 Hz, *J* = 7.4 Hz), 7.75 (ddd, 4H, *J* = 1.7 Hz, *J* = 7.6 Hz, *J* = 8.3 Hz), 7.39 (dt, 4H, *J* = 1.0 Hz, *J* = 7.5 Hz), 7.28 (dd, 4H, *J* = 0.8 Hz, *J* = 8.4 Hz), 6.27 (dd, 4H, *J* = 3.5 Hz, *J* = 6.0 Hz), 5.70 (dd, 4H, *J* = 3.6 Hz, *J* = 5.9 Hz), 3.94 (m, 8H), 3.08 (m, 4H), 2.88 (m, 4H), 2.27 (m, 4H), 1.94 (t, 8H, *J* = 3.9 Hz), 1.51 (m, 4H), −2.88 (s, 2H) ppm. ^13^C-NMR (CDCl_3_ 75 MHz) δ 158.82, 147.91, 134.91, 131.60, 129.79, 120.89, 119.85, 115.41, 114.67, 112.43, 69.41, 69.01, 68.60, 68.04 ppm. MALDI TOF *m*/*z* = 1180 (M + H^+^). UV-Vis (CHCl_3_) *λ*/nm (log(ε·M^−1^cm^−1^)) 419.5 (5.6), 513.5 (4.3), 547.0 (3.9), 589.0 (3.9), 645.0 (3.5).

Data for adjacent-*cis*-linked “basket handle” porphyrin isomer **C**: ^1^H-NMR (CDCl_3_ 400 MHz) δ 8.74 (s, 4H), 8.74 (s, 4H), 7.95 (dd, 4H, *J* = 1.7 Hz, *J* = 7.4 Hz), 7.74 (ddd, 4H, *J* = 1.8 Hz, *J* = 7.5 Hz, *J* = 8.3 Hz), 7.37 (dd, 4H, *J* = 1.0 Hz, *J* = 7.1 Hz), 7.34 (m, 4H), 6.36 (dd, 4H, *J* = 3.6 Hz, *J* = 6.0 Hz), 6.26 (dd, 4H, *J* = 3.6 Hz, *J* = 6.0 Hz), 4.11 (td, 4H, *J* = 5.6 Hz, *J* = 11.1 Hz), 4.00 (m, 4H), 3.33 (m, 4H), 3.22 (m, 8H), 2.96 (m, 8H), −2.75 (s, 2H) ppm. ^13^C-NMR (CDCl_3_ 75 MHz) δ 158.65, 148.58, 136.03, 131.63, 129.72, 121.22, 119.88, 115.65, 114.89, 112.90, 69.54, 69.28, 69.09, 68.80 ppm. MALDI TOF *m*/*z* = 1180 (M + H^+^). UV-Vis (CHCl_3_) *λ*/nm (log(ε·M^−1^cm^−1^)) 420.0 (5.6), 514.5 (4.3), 550.0 (3.8), 591.0 (3.8), 646.5 (3.4). 

#### 3.2.2. 1-Methyl-4-phenylpyridinium hexafluorophosphate (**V3**)

4-Phenylpyridine (200 mg, 1.2 mmol) was stirred with an excess of methyl iodide (0.5 mL) in acetonitrile (5 mL) for 48 h. Diethyl ether (5 mL) was added, the resulting precipitate was removed by filtration, washed with diethyl ether, and dried under vacuum. The product was dissolved in a minimal amount of water, and this solution was then added to a saturated aqueous NH_4_PF_6_ solution to yield, after filtration, washing with water and drying under vacuum, 100 mg (25%) of **V3** as a white solid. ^1^H-NMR (CDCl_3_/CD_3_CN 1:1 (*v*/*v*), 400 MHz): δ 8.61 (d, 2H, *J* = 6.3 Hz), 8.22 (d, 2H, *J* = 6.3 Hz), 7.9 (d, 2H, *J* = 7.3 Hz), 7.67–7.62 (m, 3H), 4.32 (s, 3H) ppm. ^13^C-NMR (CDCl_3_/CD_3_CN 1:1 (*v*/*v*), 75 MHz): δ 155.92, 144.61, 131.94, 129.41, 127.52, 124.47, 116.54, 47.16 ppm.

### 3.3. X-ray Analysis of S

A single crystal was mounted in air on a glass fibre. Intensity data were collected at −65 °C. A Nonius KappaCCD single-crystal diffractometer (manufacturer, city, country) was used (φ and ω scan mode) using graphite monochromated Mo-Kα radiation. Unit cell dimensions were determined from the angular setting of 227 reflections. Intensity data were corrected for Lorentz and polarization effects. SADABS multiscan correction [61] was applied. The structure was solved by the program CRUNCH [62] and was refined with standard methods using SHELXL97 [63]) with anisotropic parameters for the non-hydrogen atoms. All hydrogens were placed at calculated positions and were refined riding on the parent atoms. A structure determination summary is given in Table 5. CCDC 988037 contains the supplementary crystallographic data for this paper [64]. 

### 3.4. Determination of Association Constants

The association constants were determined by means of fluorescence- and ^1^H-NMR titrations. For the 1:2 binding of **S** to **V1** the experimental error was quite large because of uncertainties involved with determining two association constants from one single binding experiment. The fluorescence titrations giving association constants *K*_SV1_ and *K*_CV1_ were performed once, and although very good fits were obtained for the individual experiments, the experimental error was chosen on the conservative side (30%). Experimental errors evolving from weighing small quantities of receptor and potential atropisomerization during the experiment could not be fully excluded (all effort was taken to prevent this phenomenon: both **S** and **C** were purified directly before the binding studies, analyzed using ^1^H-NMR, and kept cold for as long as possible before the binding experiment to prevent the switching). For individual titration data, see below.

**Table 5:** Crystallographic data for isomer **S**.

Crystal Property	Value
Identification code	CLIP10
Crystal colour	dark purple
Crystal shape	rough thick platelet
Crystal size	0.21 × 0.20 × 0.05 mm
Empirical formula	C_72_ H_66_ N_4_ O_12_
Formula weight	1179.29
Temperature	208(2) K
Radiation/Wavelength	MoKα (graphite monochromated)/0.71073 Å
Crystal system, space group	Monoclinic, P2_1_/a
Unit cell dimensions	a, alp = 12.9808(4) Å, 90°
44169 reflections	b, bet = 14.6770(9) Å, 105.642(4)°
1.900 < theta < 25.000	c, gam = 16.2856(12) Å, 90°
Volume	2987.8(3) Å^3^
Z, Calculated density	2, 1.311 Mg/m^3^
Absorption coefficient	0.090 mm^−1^
Diffractometer/scan	Nonius KappaCCD with area detector φ and ω scan
F(000)	1244
Theta range for data collection	1.90 to 27.50°.
Index ranges	−16 <= h<= 16, −18 <= k <= 18, −21 <= l <= 21
Reflections collected/unique	50597/6846 [R(int) = 0.0684]
Reflections observed	4082 ([Io > 2σ(Io)])
Completeness to 2theta = 25.00	95.7%
Absorption correction	SADABS multiscan correction (Sheldrick,1996)
Refinement method	Full-matrix least-squares on F^2^
Computing	SHELXL-97 (Sheldrick, 1997)
Data/restraints/parameters	6846/0/398
Goodness-of-fit on F^2	1.084
SHELXL-97 weight parameters	0.033600 2.925100
Final R indices [I > 2sigma(I)]	R1 = 0.0788, wR2 = 0.1286
R indices (all data)	R1 = 0.1434, wR2 = 0.1485
Largest diff. peak and hole	0.871 and −0.257 e.Å^−3^

The association constants for the 1:1 complex formation between the different isomers and viologen derivatives were determined according to standard fitting procedures. The association constants for the 1:2 complex formation between **S** and **V1** in the ^1^H-NMR titration experiment were fitted with the use of Mathematica^®^, in which the association constant *K*_SV_ was varied and the fit provided the value of *K*_VSV_. In this paper the values for *K*_SV_ and *K*_VSV_ of the optimized fit are given. For a detailed description of the procedure we refer to the Supplementary Material. 

The assumption that at lower concentrations the binding of **V1** to **S** can be fitted with the use of a 1:1 binding isotherm can also be shown analytically. In a titration of **S** with a viologen **V** in which 1:2 complex formation is allowed both the 1:1 complex **SV** and the 1:2 complex **VSV** can form. The overall binding process in the titration is therefore: **S** + 2**V** **↔** **SV** + 18**VSV**. The association constants for the 1:1 (*K*_SV_) and the 1:2 complex formation (*K*_VSV_) are presented in Equations (5) and (6).


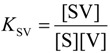
(5)


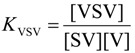
(6)

From the apparent association constant, which is given in Equation (7), it becomes clear that this constant, which describes the equilibrium of the combined 1:1 and 1:2 complexes, changes upon increasing the concentration of **V**. There is, however, a certain concentration regime in which the deviations between Equations (5) and (7) are very marginal. As long as *K*_VSV_·[**V**] is small, the overall binding process does not deviate from a 1:1 binding process. At the experimental concentrations, the maximum concentration of **V1** ([**V**]_o_) was never higher than 1 × 10^−4^ M and the concentration of **S** ([S]_o_) was 1 × 10^−6^ M. Because of this excess of **V1** it can be safely stated that [**V**]_o_ ≈ [**V**]. Assuming an association constant *K*_VSV_ of 600 M^−1^ as derived from the ^1^H-NMR titration, it becomes clear that the apparent association constant deviates only 6% from the 1:1 binding behavior at the final point of the titration (*K*_VSV_·[**V**] = 0.06), whereas in the initial part of the titration, in which lower concentrations of **V** are present, this deviation will be even smaller. Since this error is by no means larger than the experimental error in the chosen concentration regime of the titration experiment, accurate fits can be obtained with the help of 1:1 binding isotherms.


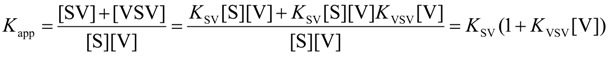
(7)

## 4. Conclusions

We have shown that the presence of viologen guests has a large influence on the thermodynamics and kinetics of the atropisomerization reaction between an adjacent-*cis*-linked and an adjacent-*trans*-linked “basket handle” porphyrin. The thermodynamic product of the guest-induced switching process strongly depends on the relative affinities of the individual isomers for the different viologen guests. In all cases, the switching experiments were in very good agreement with theoretical predictions based on the individual equilibrium constants. The kinetic studies of the switching process revealed that the presence of viologen derivatives actually enhances the rate of the switching process. It is suggested that this enhancement is caused by favorable interactions between the positively charged guest and the crown ethers of the receptor in the process of switching, which stabilize the transition state energies according to a mechanism similar to those proposed by Warshel [65,66] for the stabilization of transition states in enzymatic systems. Although the rate enhancement factor of 4 observed in this switching process is not even close to the rate enhancement observed for natural enzyme systems, which are generally many orders of magnitude larger, the cooperative action of many of these stabilizing interactions could well account for a substantial part of the enzyme proficiencies.

## Supplementary Materials

Supplementary materials can be accessed at: http://www.mdpi.com/1420-3049/19/4/5278/s1.
