# Supplementary material for: Thermodynamics and Kinetics of Guest-Induced Switching between “Basket Handle” Porphyrin Isomers
Source: Molecules. 2014 Apr 23;19(4):5278–300. doi: 10.3390/molecules19045278 (PMC6271905; doi:10.3390/molecules19045278)
Supplement: Supplementary file 1 [file molecules-19-05278-s001.pdf]

Supplementary Materials

Detailed Procedures for the Determination of Association Constants

In the 1:2 ^{1}H-NMR titration experiment between **S** and **V1** there are 4 unknowns: K_{SV}, K_{VSV}, δ_{SV}, δ_{VSV}. In which δ_{SV} is the chemical shift of a proton resonance in the 1:1 complex between **S** and **V1** and δ_{VSV} is the chemical shift of a proton resonance in the 1:2 complex between **S** and **V1**. We found no computer program powerful enough to fit a cubic equation describing 1:2 binding to the data and extract the 4 unknowns with accuracy from the single binding isotherm obtained from the ^{1}H-NMR titration. For this reason we followed an indirect and rather elaborate procedure to obtain the most accurate values for K_{SV} and K_{VSV}.

The 1:2 binding equation was programmed in Mathematica®. Random values of K_{SV} and K_{VSV} were entered and the evolution of [**S**] and complexes [**SV**] and [**VSV**] at the experimental concentrations of the ^{1}H-NMR experiment were calculated.

Using the known values of $[\mathbf{S}]_0$, $[\mathbf{V}]_0$ and δ_S and the by Mathematica® calculated values of [**S**], [**V**], and [**VSV**] at the programmed association constant, the experimentally obtained binding curve from the ^{1}H-NMR titration (δ_{obs}) could be simply fitted to the following equation

$$\partial_{obs} = \frac{\partial_S \cdot [\boldsymbol{S}] + \partial_{VS} \cdot [\boldsymbol{SV}] + \partial_{VSV} \cdot [\boldsymbol{VSV}]}{[\boldsymbol{S}]_0} \quad (1)$$

This provided the complex chemical shifts δ_{VS} and δ_{VSV} as well as a reduced Chi squared value revealing the accuracy of the fit.

By repeating this procedure while changing the into Mathematica imported values of K_{SV} and K_{VSV} a plot of reduced Chi squared values versus K_{SV} and K_{VSV} was obtained. From this landscape (Figure S1), the absolute minimum in reduced Chi squared value, and hence the best fit to the binding model could be found: $K_{SV} = 12842$ M^{-1}, $K_{VSV} = 604$ M^{-1}.

Figure S1. Plot of reduced Chi squared values versus K_{SV} and K_{VSV}.

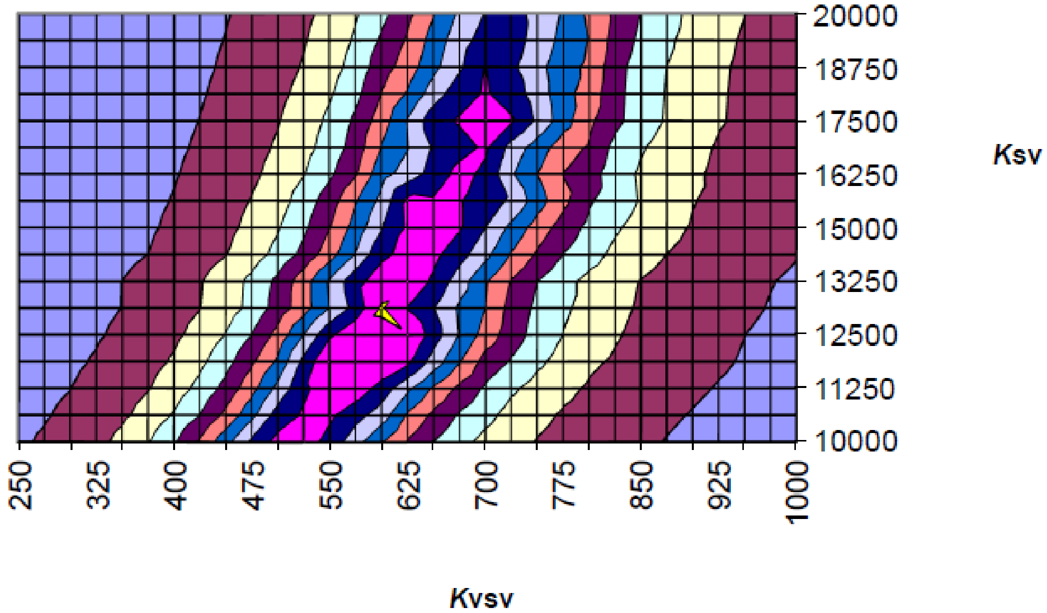

S + V1 (K_{SV} and K_{VSV}):

Figure S2. NMR titration at 298 K. ^{1}H-NMR Spectra of **S** in the presence of from bottom to top increasing quantities of **V1**.

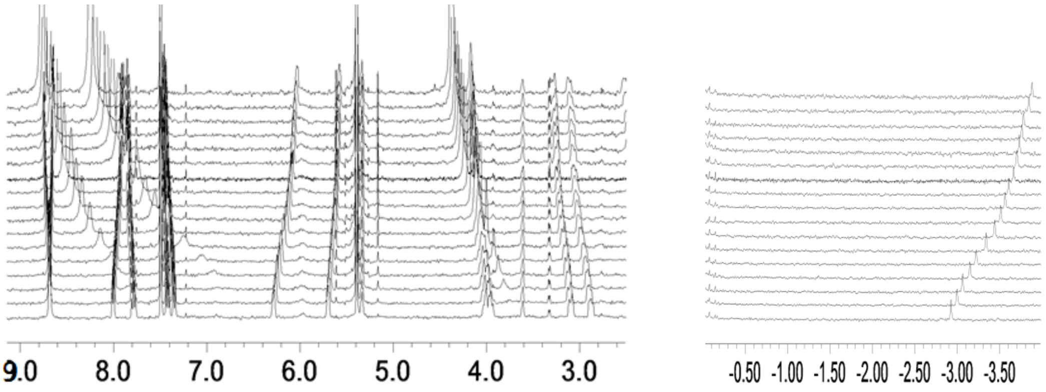

Figure S3. Binding curve of the titration between **S** and **V1** and the fits to 1:1 and 2:1 binding models.

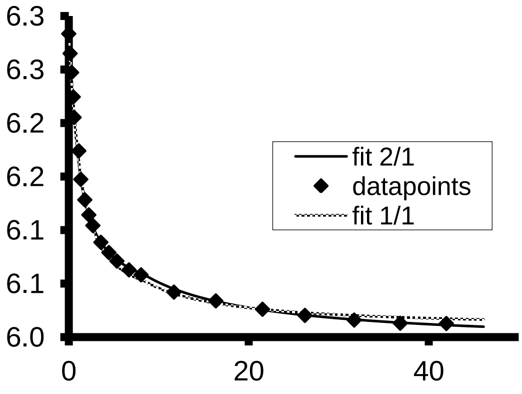

Figure S4. Fluorescence Titrations. Left: Drop in Fluorescence upon addition of **V1** to **S**. Right: normalized fluorescence emission of **S** upon addition of equivalents of **V1** with fits at different temperatures. (K_{SV} could be determined because at micromolar concentrations the 1:2 binding (K_{VSV}) is too low to significantly affect the experimental binding isotherm).

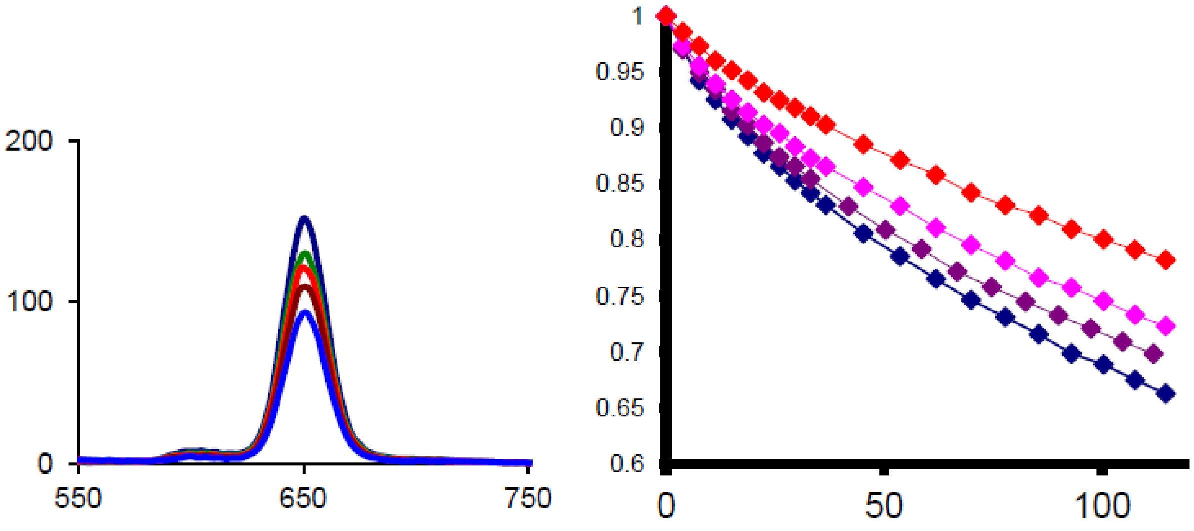

Table S1. Data Fluorescence Titrations.

T (K)	K_{SV}	*1/T*	*Ln*(K_{SV})
293	1.7×10^4	0.003413	9.740969
298	1.4×10^4	0.003356	9.546813
303	1.1×10^4	0.003300	9.341369
313	7.5×10^3	0.003195	8.922658

Figure S5. C + **V1** (K_a): ^{1}H-NMR titration. ^{1}H-NMR Spectra of **C** in the presence of from bottom to top increasing quantities of **V1**.

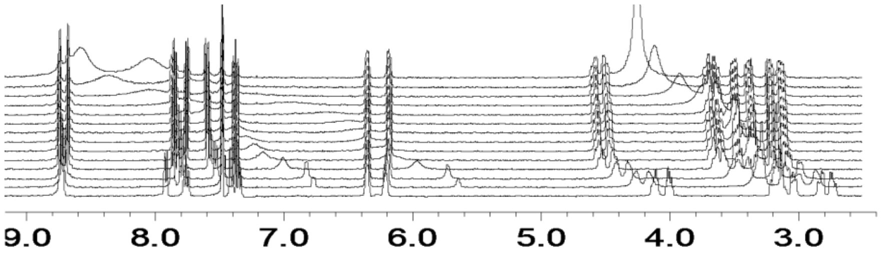

Figure S6. C + **V1** (K_a): Fit of the NMR titration: Curve too steep to determine very accurately. (Association constant too high to determine accurately at milimolar concentrations. $K_{CV1} > 1 \times 10^5$ M^{-1}).

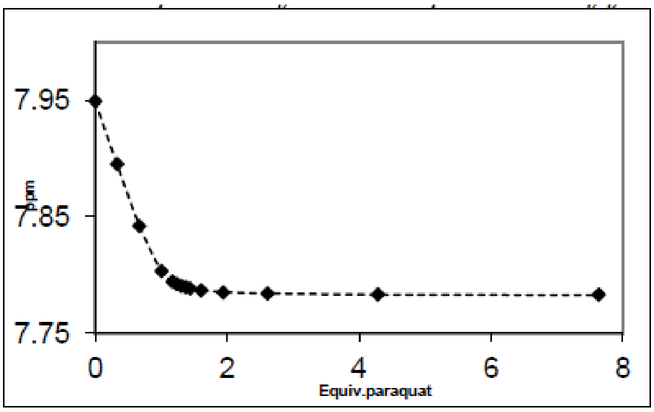

Figure S7. C + V1 (Ka): Fluorescence Titrations. **Left**: Drop in Fluorescence upon addition of V1 to C. **Right**: normalized fluorescence emission of C upon addition of equivalents of V1 with fits at different temperatures.

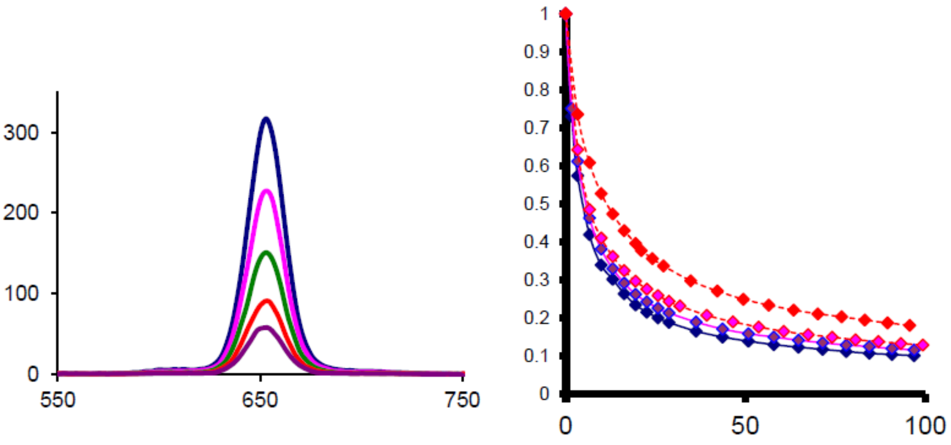

Table S2. Data Fluorescence titrations.

T (K)	K_{CV}	*1/T*	$Ln(K_{CV})$
293	3.4×10^5	0.003413	12.737
298	3.0×10^5	0.003356	12.612
303	2.7×10^5	0.003300	12.506
313	1.6×10^5	0.003195	11.951

Figure S8. C + V2 (K_{CV2}): ^{1}H-NMR titration.

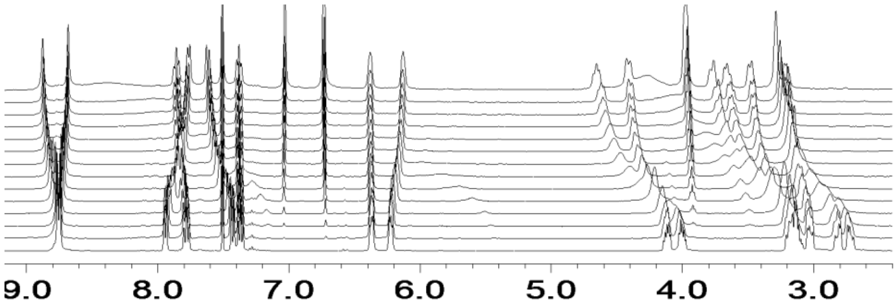

Figure S9. Curve fitting of 1H-NMR titration data of binding between C and V2 (left H-8, right β pyrrole).

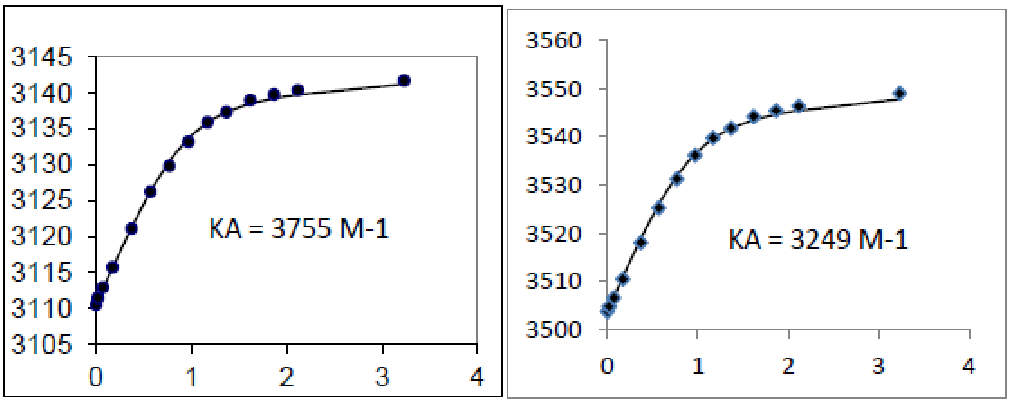

Mathematica: Example of 2:1 binding model as written into Mathematica which provides the values of the free host, free guest, the 1:1 and 1:2 complex at different values of K1, K2, total host and total guest.

```
ClearAll["Global`*"]
Clear[anul,bnul,cnul,k1,k2,k3,k4,a,b,c,anul,bnul,cnul,ac,ab,abc,abb]
k1 = 2*^4;
k2 = 1*^3;

anul = 1*^-4;
(*cnul = 2/10000;*) (*wordt nu niet gebruikt als constante, maar als variabele*)
cstart = 0.001*anul; (*kun je veranderen voor andere punten*)
cstop = 50*anul;   (*kun je veranderen voor andere punten*)
caantalpunt = 200;   (*kun je veranderen voor meer punten*)
```

```
cinterval = (cstop-cstart)/caantalpunt; (*afblijven*)
cresultaat = Table[0,{caantalpunt},{4}];
cnultable = Table[0,{caantalpunt},{1}];

goedeoplossing = {0,0,0,0};
cpunt = 1;
While[cpunt< = caantalpunt,{goedeoplossing = {0,0,0,0},Clear[cnul,a,b,ab,abb],cnul =
cstart+cinterval*(cpunt-1),
    Alex = NSolve[{k1□ab/(a*b), k2□abb/(ab*b), anul□a+ab+abb, cnul□b+ab+abb},{a,b,ab, abb}],
    teller = 1;While [teller<Length[Alex]+1,{
        If[Positive[a] /.Alex[[teller]],
          If[Positive[b]/.Alex[[teller]],
            If[Positive[ab]/.Alex[[teller]],
              If[Positive[abb]/.Alex[[teller]],
                {goedeoplossing[[1]] = a/.Alex[[teller]] , goedeoplossing[[2]] =
b/.Alex[[teller]];goedeoplossing[[3]] = ab/.Alex[[teller]];goedeoplossing[[4]] = abb/.Alex[[teller]]}]
              ]
            ]
          ]
        ,teller = teller+1}],
    cresultaat[[cpunt]] = goedeoplossing,
    cnultable[[cpunt]] = cnul,
    cpunt = cpunt+1}
  ]
cresultaat
cnultable
cplottable = Table[0,{caantalpunt},{2}]
plotteller = 1;
resulttable = Table[0,{caantalpunt},{5}]
resulttable = Transpose[Prepend[Transpose[cresultaat], cnultable]]
NumberForm[resulttable,NumberFormat→(#1"E"#3&)];

(*resultaat wordt geexporteerd als: cnul, a, b, ab, abb*)
SetDirectory["C:\Documents and Settings\Alexander\My Documents\Promotie\Theoretical
models\2to1"];                    (*hier wordt alles neergezet*)
filename = "two to one.csv";                                        (*maak file met Mijn
Computer en dan hier naam invullen*)
Export[filename,resulttable];                  (* Shift Enter om te runnen*)
```
